# Topology-aware scalable resource management in multi-hop dense networks

**DOI:** 10.1016/j.heliyon.2024.e37490

**Published:** 2024-09-10

**Authors:** David Carrascal, Elisa Rojas, Juan A. Carral, Isaias Martinez-Yelmo, Joaquin Alvarez-Horcajo

**Affiliations:** Universidad de Alcalá, 28801 Alcala de Henares, Spain

**Keywords:** Network resource management, Collaborative edge computing, Fog computing, Microgrids, Logistics, Routing

## Abstract

The current society is becoming increasingly interconnected and hyper-connected. Communication networks are advancing, as well as logistics networks, or even networks for the transportation and distribution of natural resources. One of the key benefits of the evolution of these networks is to bring consumers closer to the source of a resource or service. However, this is not a straightforward task, particularly since networks near final users are usually shaped by heterogeneous nodes, sometimes even in very dense scenarios, which may demand or offer a resource at any given moment. In this paper, we present DEN2NE, a novel algorithm designed for the automatic distribution and reallocation of resources in distributed environments. The algorithm has been implemented with six different criteria in order to adapt it to the specific use case under consideration. The results obtained from DEN2DE are promising, owing to its adaptability and its average execution time, which follows a linear distribution in relation to the topology size.

## 1 Introduction

In the contemporary era, globalization and technological advancements have fostered profound global interconnectivity in various aspects, such as communication, logistics, or the exploitation of natural resources. All these advancements, encompassed under the concept of the Internet of Everything (IoE) [5], are characterized by the presence of dense networks and heterogeneous nodes, each seeking for diverse resources based on their specific needs and capabilities.

For instance, concerning energy management, the implementation of smart grids [9] has optimized the utilization of locally generated energy, promoting self-compensation and efficiency in its distribution. Similarly, natural gas networks [78] necessitate efficient mechanisms for gas flow control [137] to interconnect consumers and producers. In the service sector, optimization of delivery routes has proven essential, both in logistics [80] and in food delivery applications [66], [84]. In the communications field, the expansion of the Internet of Things (IoT) [6], [119] and the upcoming generation of 6G networks have strengthened the focus on device interconnection [58], relying on enabling technologies such as Software-Defined Networking (SDN) [34] for logically centralized and flexible management. Within the 6G ecosystem, fog/edge computing environments might need to exchange computing capacity [12], requiring the application of different algorithms [124] and distributed strategies [13] to ensure proper cooperation among nodes [123]. The increasing density and heterogeneity of these nodes (often constrained in energy and/or computation) pose a significant challenge [102], as differences in capabilities and requirements hinder efficient resource management and, at the same time, need collaborative mechanisms to maximize performance. To address this complexity, many related works implement optimization algorithms [69], [27] that facilitate informed decision-making in resource allocation, thus ensuring a beneficial and equitable exchange among the diverse actors in these interconnected networks.

In this manuscript, we introduce Distributed ENergy ENvironments and NEtworks (DEN2NE), a novel algorithm for the distribution and automatic reallocation of resources in distributed environments. Although initially conceived for the distribution of energy among prosumers in smart grids, DEN2NE can be applied to any distributed environment where nodes need to exchange resources or collaborate on tasks with common objectives. A typical example is fog computing networks [12], where edge devices often have memory or energy limitations [56], making task redistribution crucial and requiring timely execution.

### 1.1 Contributions of our proposal

The main contribution of DEN2NE is that it is an algorithm capable of discovering the nodes and associated resources in multi-hop dense networks, subsequently sketching an efficient distribution scheme. It achieves this in an effective and scalable manner, taking the topology into account. More specifically, and in comparison with the state of the art (as analyzed in Section 2), this contribution could be divided into the following aspects:

• Discovery of meshed multi-hop topologies through a generalized hierarchical labeling assignment procedure, versatile enough for any type of network.
• Discovery of one or more paths from each network node to the topology sink, which permits establishing backup paths in the event of node failures.
• Development of a resource assignment procedure tailored for multi-hop dense networks, while granting scalability (low complexity and fast convergence time).
• Design of six different criteria enabling the adaptability of the algorithm to diverse types of networked scenarios based on the resource to be balanced.

From these contributions, we could highlight the versatility, resiliency and scalability features of DEN2NE, while considering multi-hop networks, which is a particularly novel approach in the field.

### 1.2 Article organization

The manuscript is structured as follows. Section 2 presents the related work, divided into two parts: one analyzing the closest resource management works to ours and comparing them in a table, and another summarizing the routing approaches followed for the design of DEN2NE.

Section 3 introduces the algorithm and its three core design principles, along with its terminology, phases, and sample operational criteria.

Section 4 details the algorithm's implementation, followed by Section 5, in which the algorithm's performance is assessed, also in terms of convergence time, with different random topologies.

Subsequently, after examining the algorithm's functionality and its evaluation, the discussion follows in Section 6, which also outlines use cases and fields of application to highlight the versatility of DEN2NE, concluding the paper with findings in Section 7.

## 2 Related work

Our literature analysis exhibits two main research topics in relation to our proposal (whose contributions are stated in Section 1.1): resource management in dense networks, and scalable and resilient label-based routing in multi-hop networks. In the following paragraphs, we explore the main related works for each one.

### 2.1 Resource management in dense networks

Resource management has been extensively addressed in fog and edge computing scenarios [2], [54], [89], [12], and it can be typically classified in five phases: estimation, discovery, monitoring, orchestration, and allocation [12]. In our algorithm definition, we particularly focus on two of them: discovery and allocation.

In this field, the majority of related works leverage Artificial Intelligence (AI)/Machine Learning (ML) as decision-making algorithms [65], [8], [82], [14], [110]. In the case of dense networks (usually at the edge), some research items define Collaborative Edge Computing (CEC) mechanisms or task offloading among the fog nodes in the network [114], [92], [91], [93], [129], [79], [128], [74], even considering Device-to-Device (D2D) communications at the far edge [55], [96], [62], [22], [42], [43], [90], [28].

In contrast, multi-hop resource management (using nodes as relays for the exchange of computing or radio resources) has recently started to be explored in the literature [130], [48], [30], [52], [61], [21], [117], [4], [47], [29], [57], [10], [41], [72], [85], [87], [88], [94], [109], [131], [134], [135], [136] (the majority of these works were published during the last year), but focusing on very specific scenarios and use cases (e.g. Unmanned Aerial Vehicle (UAV), satellite, vehicular or industrial networks). For instance, some of the particularities of these works include limiting the amount of hops, relying on estimation or prediction to consider network updates or mobility, or even directly neglecting dynamic changes of networks.

Additionally, few works consider scalability and/or resiliency as a design parameter. For example, energy consumption is contemplated in some approaches [130], [122], [126], [7], [62], but excluding the actual complexity of the algorithm in terms of energy efficiency or scalability for dense networks.

Finally, it is important to highlight that these works are mainly designed for fog and edge computing scenarios. Related works to other types of networks (such as smart grids) are even scarcer [20], [63].

For a quick overview of our proposal in comparison with the state of the art, Table 1 summarizes the closest works and details three main features: convergence time, complexity and versatility, which we consider key design objectives of DEN2NE (as later defined in Section 3). Converge time indicates the amount of time required for the algorithm to converge in practice (but most works do not even measure this aspect); complexity refers to implementation (centralized or decentralized/distributed), associated technique and scalability (O, although many most of them do not measure it and some values were inferred in our analysis and probably not precise); and finally versatility states the associated application or use cases, as well as the network type.

**Table 1:** Summary of the closest related works to DEN2NE, revising three specific features: convergence time, complexity and versatility.

Article	Year	Convergence time	Complexity	Versatility
Hong et al. [48]	2019	—	Centralized (Game theory) / $O(n^{2})$	Edge computing (Industrial networks)
Deng et al. [30]	2020	—	Centralized / —	Edge computing (Vehicular networks)
Hussain et al. [52]	2021	—	Centralized (Optimization) / —	Edge computing (Vehicular networks)
Kaneva et al. [61]	2021	—	Centralized (AI) / —	Edge computing
Chen et al. [21]	2021	—	Decentralized / —	Edge computing
Tong et al. [117]	2022	—	Centralized (AI + Optimization)) / —	Edge computing (UAV networks)
Ahmad et al. [4]	2023	—	Centralized (AI) / $O(n^{2})$	Edge computing (UAV networks)
Hoa et al. [47]	2023	—	Centralized (AI) / —	Edge computing (UAV networks)
Deng et al. [29]	2023	—	Centralized (Optimization + Matching theory) / —	Edge computing
Zhang et al. [132]	2023	—	Centralized (Optimization) / —	Edge computing (Satellite networks)
Ma et al. [72]	2024	15 s (20 nodes)	Centralized (Optimization)/ $O(n^{2})$	Edge computing (IoT networks)
Huang et al. [50]	2024	<40 CPU cycles (200 nodes)	Centralized (Hierarchical minority game + Routing)/ O(n)	Edge computing (IoT networks)
Zhao et al. [134]	2024	200 s (100 nodes)	Centralized (Optimization)/ $O(n^{2})$	Edge computing (Satellite networks)
Janji et al. [57]	2023	—	Centralized (Optimization) / —	Radio coverage (UAV networks)
Gures et al. [41]	2024	—	Centralized (Optimization) / —	Radio coverage (UAV networks)
Nikooroo et al. [85]	2024	—	Centralized (Optimization) / —	Radio coverage (UAV networks)
Pan et al. [87]	2024	—	Centralized (Optimization) / —	Radio coverage (UAV networks)
Panigrahi et al. [88]	2024	—	Centralized (Optimization) / —	Radio coverage (5G)
Shang et al. [109]	2024	—	Centralized (Optimization) / —	Radio coverage (6G)
Qin et al. [94]	2024	—	Centralized (AI) / —	Radio coverage (Satellite networks)
Zhang et al. [131]	2024	—	Decentralized (AI + Routing) / —	Radio coverage (Wireless ad hoc networks)
Zhao et al. [135]	2024	—	Centralized (AI) / —	Edge computing (Vehicular networks)
Zhao et al. [136]	2024	—	Decentralized (AI + Routing) / —	Edge computing
Alvarez-Horcajo et al. [10]	2024	<6 s (9 nodes)	Decentralized (Routing) / O(n)∼$O(n^{2})$	Edge computing
Li et al. [63]	2024	<40 s (average)	Centralized (Optimization + Routing) / —	Smart grids (Power IoT)

In conclusion, we consider the closest works to our algorithm are Ma et al. [72], Huang et al. [50], and Zhao et al. [134]. However, to the best of our knowledge, our proposal yields better results regarding convergence time and scalability, and it is more versatile, which may possibly be due to the fact that no proposal simultaneously considers convergence time, complexity, and versatility as their design foundations.

### 2.2 Scalable and resilient label-based routing in multi-hop networks

As an inspiration for the design objectives of DEN2NE, we considered existing routing approaches for scalable and resilient communication. In fact, the problem statement of resource management could be defined as a routing problem, in which sources and destinations are undefined (differently from communication networks, in which source and destination/s are clearly defined prior to path establishment). When searching for scalable routing mechanisms in multi-hop networks, the implementation of label- or address-based source routing approaches [36] can substantially benefit scalability, as the need for computational resources and energy can be drastically reduced [60].

This type of routing is commonly implemented in computer networks by leveraging the local Medium Access Control (MAC) address space structure [1]. For example, in data center networks, many works leverage address-based source routing, like PortLand [86], VL2 [37], among others [40], [49], [95], [100], [76], [26], [133], [108], [36], all of which require specific network topologies (Clos, tree-based or other hierarchical shapes), and some can be even generalized for any type of network [3], [105], [60], [121]. Additionally, address-based source routing is also presented in proposals in relation with SDN in-band control (Amaru [68]), IoT networks (IoTorii [99]) or reliable multipath routing (M-PolKA [38], [18]).

For most of these works, resiliency is an indirect consequence of source routing, as in some cases multiple paths can be inferred, hence resulting in backup paths that can protect networks from failures. Nevertheless, some works specifically focus on enhancing resiliency via rerouting implemented with segment routing (a type of source routing) [81], [23].

Following this label- or address-based approach could improve the current scalability and resiliency of existing resource management designs. Therefore, our algorithm design is founded on it.

## 3 Algorithm definition

To explain the objectives and design principles of our algorithm, let us first explain their context and motivation with a network composed of multiple nodes that are resource-constrained and/or heterogeneous, in which one or more tasks need to be completed in time. Each of these tasks cannot be completed by a sole node alone. Therefore, in these networks, nodes should necessarily delegate and cooperate to finalize their tasks together but, at the same time, they should take into account each other's needs so that the redistribution is fair enough.

**Figure 1:**
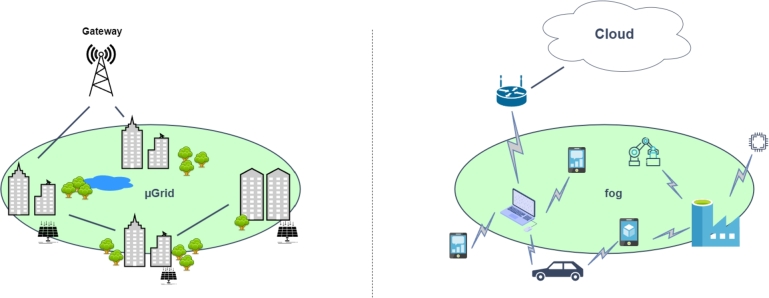
Two examples of networks that would leverage DEN2NE for resource balancing.

Fig. 1 illustrates two examples of these networks. On the left side, a microgrid [45] is depicted. Microgrids systems are energy networks that involve a set of energy *prosumers* (nodes that can be both producers and consumers of electricity, usually because they have a renewable energy source installed, like a solar panel). These systems need to redistribute surplus production among other consumers, and this energy distribution should occur as close as possible to the source to minimize energy losses when transmitted. On the right side, a fog computing [11] network is represented, which is comprised of diverse IoT devices, such as laptops, mobile phones, cars, or industrial robots. In this latter scenario, one IoT node might need to compute a task, and offloading it among other close devices with available computing resources might accelerate the finalization of that task. As it can be observed, both deployments require an analogous algorithm to share one or more actions/tasks to be finalized (e.g., energy production, task computation, etc.). Additionally, both exemplify a portion of a much bigger network, which implies they have a connection (i.e. gateway) to the rest of that bigger network. For instance, a microgrid could use the gateway to send or receive energy to the rest of the smart grid deployment, and the same happens with the fog computing environment, which could delegate the finalization of the task to the cloud in the worst-case scenario (that is, when all nodes are too loaded to compute it locally).

Accordingly, the main objective of our algorithm is to efficiently redistribute resources among any type of connected nodes. For this purpose, any source node might delegate a resource to any other destination node. In this regard, the problem statement resembles a routing problem with a difference: when routing, there is a piece of information that should be delivered from one specific source to one specific destination and only the route needs to be calculated; while in this case, there is still a defined source and information, but the potential destinations could be any and should be decided together with the route to be followed by the information. In other words, one resource could be shared among one or more destination nodes and that decision should be computed by the algorithm as well as the route to reach them.

To accomplish the previous objective, the design of the DEN2NE is grounded in three core principles:

1. **Fast convergence time**: The calculation of resource sharing decisions should be fast, that is, the algorithm should have a low convergence time. At the same time, this convergence time should not be drastically affected by the amount of available data or the number of nodes in the network, i.e., it should preferably have a linear growth with network size (O(n)). Low convergence times facilitate easy updates in high-mobility scenarios.
2. **Low complexity**: As nodes leveraging this algorithm will be usually constrained in resources (e.g., memory or energy), the algorithm should require low computational capacity to be executed, should need low amounts of memory (storage), and should imply a small number of exchanged control messages (if any).
3. **Versatility**: While the two previous aspects mainly focus on scalability, this third design criterion emphasizes on practical implementations and, how the algorithm should be versatile enough to be adapted for different networks (wired and wireless networks, smart grids, etc.), centralized/distributed approaches, etc. More specifically, the algorithm should avoid any particular environmental constraint in its design so that it can be easily reused and adapted to different and generalized scenarios.

Based on these three design principles, the IoTorii [99] routing protocol was considered as a starting point. The reason is that it accomplishes the fast convergence time and low resource consumption requirements. Even if implemented in a distributed manner, IoTorii can be easily converted into a centralized version. However, as a routing protocol, it still selects paths based on specific origin and destination nodes, so it should be modified to accomplish the objectives of our algorithm, which should not require the definition of a set of destination nodes as an input.

More specifically, DEN2NE is designed to look for the closer neighbors to which a specific task or resource could be shared. In this scenario, we assume there is always, at least, one node that is connected to *infinite* resources, so that it can serve as a network resource sink. This type of node is, in fact, what we previously called a gateway node. Gateway nodes are root nodes that can adjust remaining parts of the task or resource when local offloading is not feasible. In the following sections, the DEN2NE algorithm is described in detail.

### 3.1 Algorithm terminology

**Figure 2:**
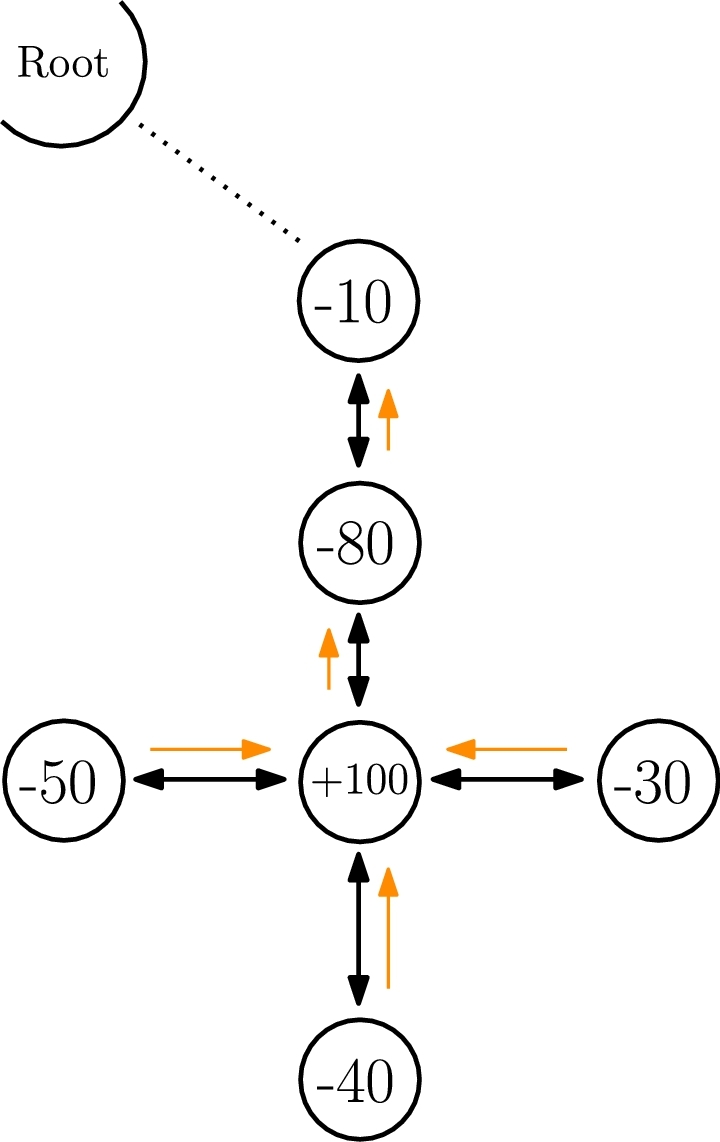
Example of resource balancing in a network with six nodes and a gateway (or root node).

Once the algorithm motivation has been introduced and before delving into its description, we will define a few terms used afterwards. For the sake of simplicity, let us focus on Fig. 2, as an example scenario of application. In this scenario, we have six ***nodes*** (depicted as circles) connected to one ***root*** node. The nodes are bidirectionally ***connected*** (either in a wired or wireless manner) as indicated by the darker bidirectional black arrows, and their numbers symbolize a ***resource*** that needs to be distributed or balanced. More specifically, that resource stands for an ***offer*** or a ***demand*** (e.g., electricity production/consumption or computational capabilities available or required). In the case of Fig. 2, negative values express a demand, while positive ones express an offer. In this regard, for instance, a demand of -80 would be compensated by an equivalent offer of +80 or, alternatively, a combination of offers that added up to +80. Therefore, the main objective of our algorithm is to compensate all resources in the network, that is, all nodes should tend towards the value zero (optimal usage), while the remainder (either excess of demand or offer) should be redirected to the root node.

Before describing the three phases of the DEN2NE algorithm (in Sections 3.2, 3.3, and 3.4, respectively), let us now think of the example in Fig. 2 to understand why we defined three phases. Considering the above-mentioned design principles, calculating all possible sharing combinations is not feasible in a reasonable time. However, on the other hand, local information might reduce convergence times and complexity, but might not be enough to optimally decide either. As an example of a bad decision, the node with a demand of -80 is only aware of its two neighbors: one offering (+100) and another demanding (-10), and it might think that the most suitable node to cover its demand (-80) is the neighbor below (+100) (in Fig. 2). However, this local decision is eventually wrong, because there are other nodes with additional demands (-50, -40, and -30), which require that offer as well and are located much further from the root node. In fact, the optimal distribution solution is illustrated with light orange arrows (which represent the direction from the requesting node to the offering one). This distribution would imply all nodes are balanced to zero and the remaining demand of -110 (sum of -50 -40 -30 +100 -80 -10) would be diverted towards the root node, capable of covering it.

Consequently, the DEN2NE algorithm calculates this resource distribution by considering information from all traversed nodes, instead of simply local information from neighbor nodes. Furthermore, it prioritizes resource distribution at the nodes furthest from the root node, sweeping the remainder (offer or demand) to the root, so that it can be compensated there by the rest of the network (e.g., the cloud or smart grid).

In the following subsections, we will explain in detail the three phases that comprise the algorithm. The first phase is devoted to explore the topology and to aggregate information from traversed nodes by using hierarchical labeling; the second selects the set of information (i.e. label), from all received, that seems closer to the optimal solution; while the third simply executes the resource balancing based on the decision made in the second phase. More specifically:

• In **Phase 1** (Subsection 3.2), we will explain the hierarchical labeling to compute routes from each node to the root node. This is inspired by the related works for scalable and resilient routing examined in Section 2.2.
• In **Phase 2** (Subsection 3.3), once all possible paths to the root node have been computed, one of them will be selected at each node based on a specific criterion. This criterion provides the versatility envisioned as a design principle, that is, we provide a sample list of six criteria so that the most suitable one is applied to each different scenario, but in practice any alternative/new criterion could be potentially applicable if required.
• In **Phase 3** (Subsection 3.4), following the selection of the active path ($ID_{active}$) in the previous phase, resource balancing is carried out based on the list of nodes represented by the selected or active labels. This phase also considers the actual conditions of the network, for instance, if the nodes are connected via realistic links with losses.

### 3.2 Phase 1 - hierarchical labeling

**Figure 3:**
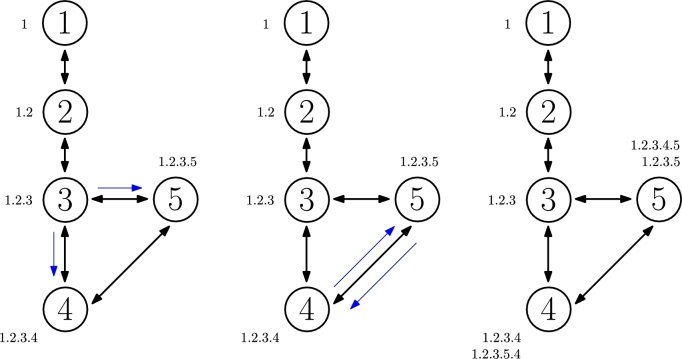
Hierarchical labeling procedure.

The DEN2NE algorithm starts by locating the relative positions of each node in the network. For this purpose, a hierarchical labeling is performed from each gateway or root node existing in the deployment. As a centralized algorithm, each node will have a unique numerical identifier (ID), and will obtain a label or hierarchical ID in the process. The procedure starts at the root node, which in Fig. 3 has ID 1 and one connected neighbor, with ID 2. To hierarchically correlate both, the algorithm assigns the label or hierarchical ID 1 to the root and append the neighbor ID to its own, so that node 2 is assigned the label or hierarchical ID 1.2. Afterwards, the node with ID 3 will obtain label 1.2.3, and nodes with IDs 4 and 5 will be assigned labels 1.2.3.4 and 1.2.3.5, respectively. Furthermore, as the assignment continues, node with ID 4 will also obtain label 1.2.3.5.4 and node with ID 5 will obtain 1.2.3.4.5, accordingly. This labeling procedure continues until the whole network is explored and each node should obtain, at least, one hierarchical label. In practice, each label represents a route to reach the root node.

**Figure 4:**
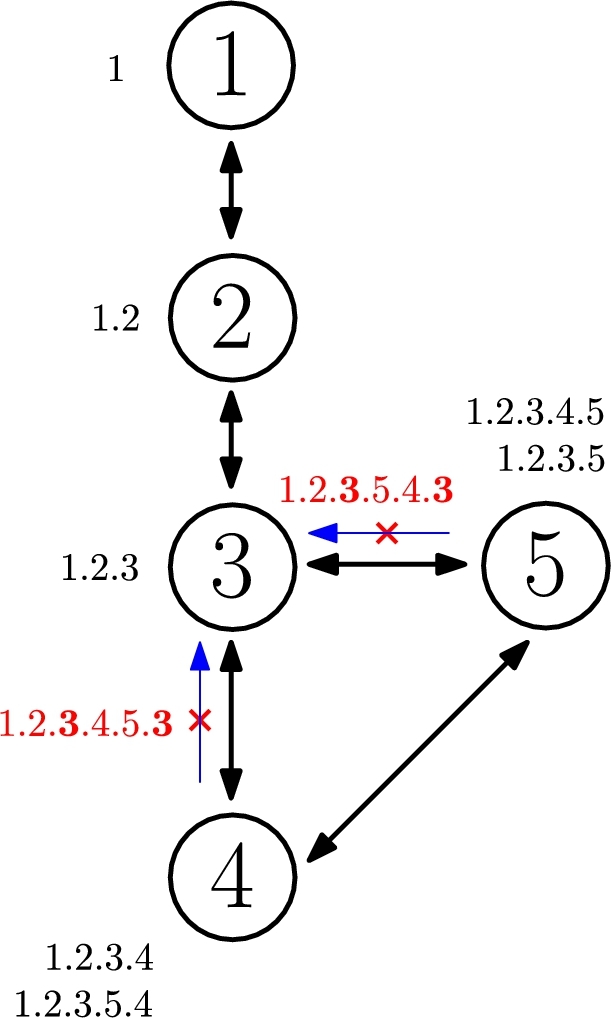
Loop avoidance during labeling.

To avoid loops during this hierarchical labeling phase, the algorithm does not assign labels to a node inherited from itself. This can be easily foreseen as labels append the traversed nodes IDs, so no label should have two repeated IDs in it as, otherwise, it would mean a loop was generated. For instance, Fig. 4 graphically demonstrates how this action would be performed. In particular, it illustrates the next step in relation to the ones depicted in Fig. 3. When the node with ID 5 receives label 1.2.3.5.4 from node with ID 4, it could try to assign label 1.2.3.5.4.3 to node with ID 3, but this action is not performed because it contains ID 3 twice in it, which represents a network loop. Once this label is discarded, no additional labels appending from it will be propagated. Analogously, the same will happen when the node with ID 4 generates label 1.2.3.4.5.3 for node 3 too. In fact, after these steps, no additional labels will be assigned in this specific example and the algorithm procedure will have ended at this point.

As previously stated, each assigned label represents one path to the root and each node obtains one or more labels (i.e., multiple paths to the root node). Therefore, this first algorithm phase potentially calculates all possible paths from any node in the network towards the root node. This might imply thousands or even millions of routes, depending on topology connectivity. Thus, implementation-wise, this hierarchical labeling phase can be limited to learn only a set of labels and discard the rest, following certain parameters, such as path disjointness or minimum number of hops [68], [99].

Algorithm 1 summarizes the whole procedure of Phase 1, which takes as an input the network graph and the node to start the hierarchical labeling from (i.e., the root node), and it returns the graph with the corresponding assignment of labels. The algorithm traverses all nodes starting from the neighbors of the root node. Nodes might be checked more than once, up to a maximum of assigned labels (IDs_MAX) or if a loop is found (check_loop). For that reason, we could state that the complexity of the labeling phase is O(n), as it is linear with the number of nodes *n*, which are visited a maximum of IDs_MAX times, regardless of their neighbors or topology shape or size.

**Algorithm 1:**
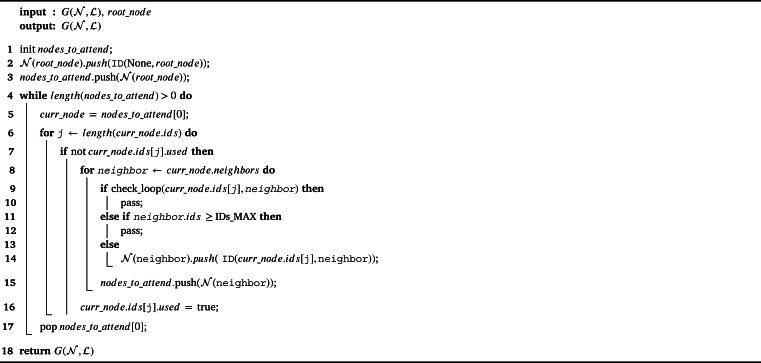
Hierarchical labeling process.

### 3.3 Phase 2 - selection of best labels or hierarchical IDs

As previously introduced, root nodes or gateways are nodes connected to other resources (energy, computation capabilities, etc.), which could be considered infinite. For this reason, when redistributing a task, nodes located further from the root are less prone to have available resources around or locally, while nodes closer to the root can easily delegate a task to the gateway for its finalization. In fact, the first phase of the algorithm provides the relative location of nodes in relation to the root, so that we can easily spot, for task offloading, what nodes should have a preference over others when deciding where to delegate a task. This greatly simplifies the process, as it creates an order for decision making.

More specifically, once the labeling procedure has finalized, all nodes can use any of the assigned labels to reach the root node, as labels implicitly represent the list of nodes to be traversed to reach the root. When only one label is associated to a node, there is only one path to the root. However, if multiple labels exist, certain criteria should be implemented to select the most appropriate route towards the root. Additionally, this selection criterion should be applied in certain order, as routes towards the root node are built together from the rest of nodes and should be coordinated.

Accordingly, the second phase of the algorithm decides where to distribute resources applying certain criteria that follows the order predefined by the labels or hierarchical IDs configured during the first phase.

As a reference, the DEN2NE algorithm provides six different criteria to select the best route for resource distribution towards the root, which are based on diverse literature works. Each criterion considers different topological aspects and characteristics of the deployment to calculate a cost for each available route, that is, each hierarchical ID assigned (Cost_ID). Afterwards, the algorithm selects the best route or active hierarchical ID (ID_active) based on the minimum cost, as indicated by Equation (1).

(1) $$\langle ID_{active}\rangle \;=\;min(Cost_{ID})$$

The six criteria are described in the following sections. Additional criteria could be envisioned, and incorporated in the algorithm, since this second phase only requires the definition of a cost function for each criterion, but it is not strictly limited by a certain criterion per se (in fact, this grants the versatility of our proposal). Furthermore, some criteria might be applicable to any type of use case, while others might be specific to certain scenarios (smart grids, fog computing, etc.). For these reasons, the criteria are explained in a simple and fast manner, so that the reader can have a reference, but DEN2NE is potentially agnostic to the criterion selected in practice.

#### 3.3.1 Criterion 1 - number of hops

This first criterion evaluates the number of hops to reach the root node, so that the hierarchical ID with a fewer number of hops is selected at each node. It is based on a classic shortest path criterion for routing in communication networks, which is called *widest-shortest path* by Ma et al. [73]. Therefore, the cost function for this criterion can be expressed as in Equation (2).

(2) $$Cost_{ID}\;=\;length(ID)\;-\;1$$

**Figure 5:**
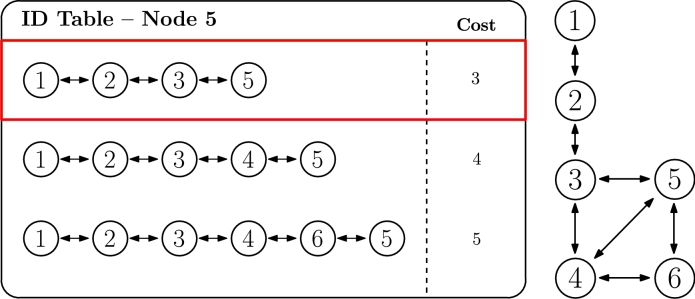
ID selection based on the number of hops criterion.

To understand it, Fig. 5 illustrates an example in which node 5 has acquired three hierarchical IDs and the three potential routes to the root (node 1) are depicted. In this scenario, the cost is calculated as the length of the ID (where the length is the number of cyphers in the hierarchical ID) minus one, which for ID 1.2.3.5 is 3=4−1, for instance, as that ID has 4 cyphers. Once the cost is calculated for every hierarchical ID, the selected ID is 1.2.3.5 in the example, according to Equation (1), as it has the lower cost, which implies fewer hops to reach the root. In case of failure or unavailability of that main route, any of the remaining ones could be used, ordered by cost.

#### 3.3.2 Criterion 2 - distance

The second criterion involves the concept of distance between nodes. This distance could be either a physical distance (for example, for microgrids deployments, in which this physical distance is well-known and constant as nodes are not mobile) or logical distance (for example, a link cost based on throughput or latency, in a network deployment). Similarly to the previous criterion, it is based on classic routing in communication networks, and it is called *shortest-distance path* by Ma et al. [73]. In this case, the cost of each ID is calculated based on the sum of the distance of the traversed links as represented by Equation (3) (at the same time, a distance could also be the inverse value of the link throughput or any other related measure).

(3) $$Cost_{ID}\;=\;\sum_{i=0}^{N_{hops}-1}d_{i}$$

**Figure 6:**
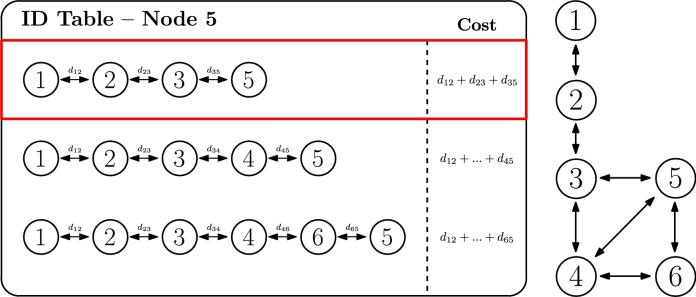
ID selection based on distance criterion.

Fig. 6 illustrates the same example as before, but applying this second criterion. In this scenario, the same ID 1.2.3.5 is selected, assuming the condition in Equation (4) is accomplished. However, any other ID could be selected, depending on the actual value of the distances, even if the route contains more hops until the root.

(4) $$\langle ID_{1}=ID_{active}\rangle \Leftrightarrow \left\{ \begin{array}{c} \left[d_{35}<(d_{34}+d_{45})\right]\\ \wedge \\ \left[d_{35}<(d_{34}+d_{46}+d_{65})\right] \end{array}\right.$$

#### 3.3.3 Criterion 3 - link losses

The third criterion is directly related to the smart grid use case (inspired by the IEE 123 node test feeder topology [104]), in which this algorithm could be applied, and it is also somehow attached to the previous criterion about distance. More specifically, it pursues the minimization of link losses caused by the transmission links. To distinguish this criterion from the previous one, we should highlight that criterion 2 usually focuses on link characteristics or capacity (e.g. link throughput or distance), while criterion 3 focuses on link degradation effect (e.g. power loss). Nevertheless, both criteria are closely related and, in fact, their formula is conceptually similar, exchanging distances by losses, as represented in Equation (5).

(5) $$Cost_{ID}\;=\;\sum_{ij}^{N_{ID}}L_{ij}\;=\;\sum_{ij}^{N_{ID}}(\frac{\alpha_{R_{ij}}\;\times \;d_{ij}}{V_{d}^{2}}\;\times \;P_{in}^{2})$$

In the specific case of smart grids, losses are calculated based on the loss models for transmission lines, which consider parameters such as: voltage ($V_{d}$), physical distance ($d_{ij}$), reflection coefficient ($\alpha_{R_{ij}}$), and input power ($P_{in}$), as indicated in Equation (6) (included in Equation (5)). As it can be observed, physical distance is one of the parameters that affect the cost, but not the only one. Thus, as we previously mentioned, both critera 2 and 3 are attached, but they might not necessarily yield the same cost result, as other factors are involved too. In other scenarios, losses could represent an analogous meaning (e.g. link reliability in a wireless for computing network environment).

(6) $$L_{ij}\;=\;\frac{\alpha_{R_{ij}}\;\times \;d_{ij}}{V_{d}^{2}}\;\times \;P_{in}^{2}$$

**Figure 7:**
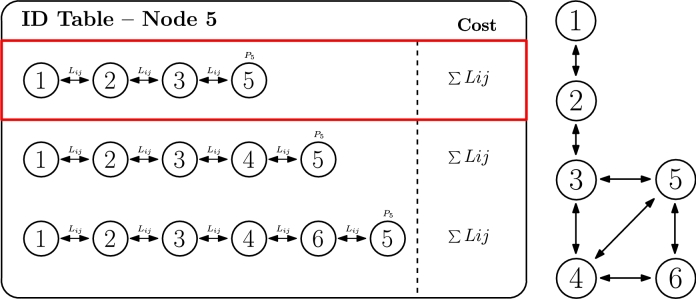
ID selection based on link losses criterion.

In the specific example of Fig. 7, the path towards the root node selected by the $ID_{active}$ is the first one (1.2.3.5), which in this case is the same as in the previous criterion. Equation (7) is accomplished for this $ID_{active}$ to be set.

(7) $$\langle ID_{1}=ID_{active}\rangle \Leftrightarrow \left\{ \begin{array}{c} \left[\sum_{\begin{array}{c} 1\leq i\leq 3\\ 2\leq j\leq 5\\ j\neq 4 \end{array}}L_{ij}<\sum_{\begin{array}{c} 1\leq i\leq 4\\ 2\leq j\leq 5 \end{array}}L_{ij}\right]\\ \wedge \\ \left[\sum_{\begin{array}{c} 1\leq i\leq 3\\ 2\leq j\leq 5\\ j\neq 4 \end{array}}L_{ij}<\sum_{\begin{array}{c} 1\leq i\leq 5\\ 2\leq j\leq 6 \end{array}}L_{ij}\right] \end{array}\right.$$

#### 3.3.4 Criterion 4 - power balance

The fourth criterion evaluates the routes with more available resources when traversed, and preferably selects them against others. Since each node will have a resource offer (positive value) or demand (negative value), adding up all values in the route will represent a total cost of the route, as defined in Equation (8). This cost is calculated by changing the sign of the sum of all powers along the path, as our objective is to minimize it. Therefore, if there are many resource offers, the sum will be a big positive value and reversing its sign will be translated into a very negative value (i.e., low cost), and vice versa. It is important to highlight that the name of this criterion comes from the smart grids use case in which resources are electrical power. However, although power offer and demand is considered in the formula, other parameters such as computing capacity in CEC environments could be analogously applied in this criterion. In fact, in the field of classic routing, it is called *shortest-widest path* by Ma et al. [73], while Gunantara et al. [39] also considers a similar metric, as the amount of power consumption per path.

(8) $$Cost_{ID}\;=\;-(\sum_{i}^{N_{ID}}P_{i})$$

**Figure 8:**
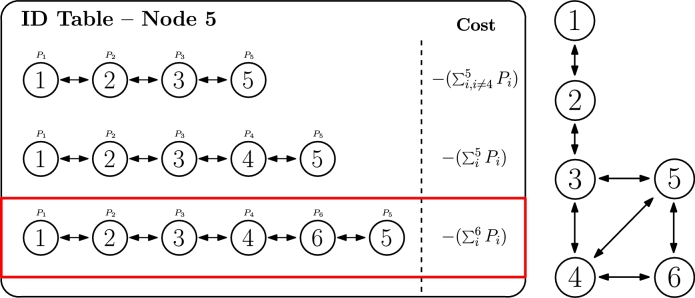
ID selection based on power balance criterion.

Once again, selecting $ID_{active}$ will be based on minimum Cost_ID. In the case of our example, as seen in Fig. 8, the selected ID is 1.2.3.4.6.5, which should accomplish Equation (9) (even if power values are not indicated).

(9) $$\langle ID_{3}=ID_{active}\rangle \Leftrightarrow \left\{ \begin{array}{c} \left[\sum_{i}^{6}P_{i}>\sum_{i}^{5}P_{i}\right]\\ \wedge \\ \left[\sum_{i}^{6}P_{i}>\sum_{i,i\neq 4}^{5}P_{i}\right] \end{array}\right.$$

#### 3.3.5 Criterion 5 - lossy power balance

The fifth criterion merges both criteria 3 and 4 [104], [73], [39], as it searches for maximising available resources in the path, while limiting the potential losses that a specific route (specially if longer than others) might convey. Once again, in the case of smart grids, it is translated into power offer and demand, subtracting the losses in the route (which follow Equation (6)), as represented in Equation (10).

(10) $$Cost_{ID}\;=\;-(\sum_{i}^{N_{ID}}P_{i}-\sum_{ij}^{N_{ID}-1}L_{ij})$$

**Figure 9:**
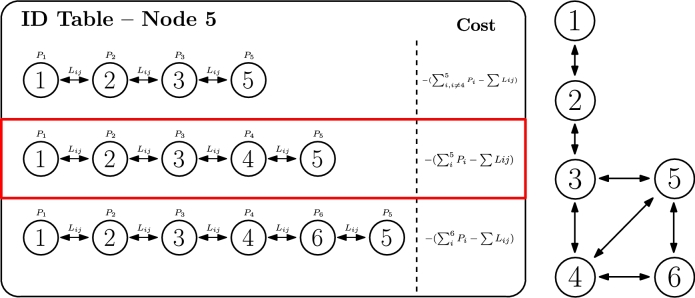
ID selection based on lossy power balance criterion.

When this criterion is applied to the example in Fig. 9, the selected path is 1.2.3.4.5, which in fact represents an intermediate decision between criterion 3 (link losses) and 4 (power balance). Accordingly, Equation (11) needs to be fulfilled so that $ID_{active}$ translates into the one in Fig. 9.

(11) $$\langle ID_{2}=ID_{active}\rangle \Leftrightarrow \left\{ \begin{array}{c} \left[(\sum_{i}^{6}P_{i}-\sum L_{ij})>(\sum_{i}^{5}P_{i}-\sum L_{ij})\right]\\ \wedge \\ \left[(\sum_{i}^{6}P_{i}-\sum L_{ij})>(\sum_{i,i\neq 4}^{5}P_{i}-\sum L_{ij})\right] \end{array}\right.$$

#### 3.3.6 Criterion 6 - weighted power balance

The sixth and last criterion represents an alternative implementation, using weights, of the ideas presented in the two previous criteria [104], [73], [39]. While criterion 5 penalizes the route based on length and other loss parameters, criterion 6 normalizes the route power capacity based on the number of traversed nodes. This criterion directly highlights the pernicious effect that criterion 4, which searchs for the biggest power balance, might have, as it might eventually look for longer paths to the root. Therefore, criterion 5 readjusts this calculation by considering potential losses as well, and criterion 6 provides a weighted power balance, so that capacity is considered but number of hops in the route too. This criterion might be particularly useful when no representation of loss can be easily portrayed. The resulting formula is represented in Equation (12).

(12) $$Cost_{ID}\;=\;-(\frac{1}{M}\sum_{i}^{N_{ID}}P_{i}),\;\;M=\frac{1}{\left(lenght(ID)\;-\;1\right)}$$

**Figure 10:**
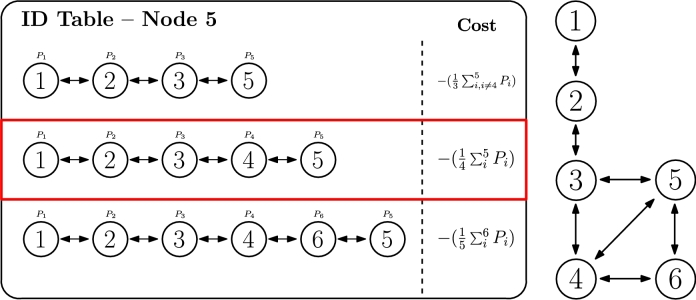
ID selection based on weighted power balance criterion.

Considering the example of Fig. 10, $ID_{active}$ is route 1.2.3.4.5, which has a lower amount of nodes than the one selected in criterion 4, as expected, but the best relation $\frac{resources}{hops}$ among all. This route accomplishes the condition represented in Equation (13).

(13) $$\langle ID_{2}=ID_{active}\rangle \Leftrightarrow \left\{ \begin{array}{c} \left[\frac{1}{4}\sum_{i}^{5}P_{i}>\frac{1}{3}\sum_{i,i\neq 4}^{5}P_{i}\right]\\ \wedge \\ \left[\frac{1}{4}\sum_{i}^{5}P_{i}>\frac{1}{5}\sum_{i}^{6}P_{i}\right] \end{array}\right.$$

### 3.4 Phase 3 - global balance

Once labels are assigned along the whole topology, certain criterion has been applied, and each node has one $ID_{active}$, the procedure to balance the available resources may start. The objective of the algorithm is to yield a global balance state by moving resources from the further nodes towards the root. This movement is implemented locally, but following a specific order based on node's $ID_{active}$, which eventually produces an overall balance of resources for all topology nodes.

To proceed, as the algorithm is centralized, it is possible to collect each node's $ID_{active}$, and order them based on length. The longer the $ID_{active}$, the further the node is logically located to reach the root.^1^ Therefore, the algorithm starts balancing the load from the longest $ID_{active}$, which is associated to one specific node of the network. In case two or more $ID_{active}$ have the same length, one of them will be picked at random.

Once nodes are ordered based on their $ID_{active}$, each node is visited just once by the algorithm to balance the resource with its predecessor node in the ordered list represented by the $ID_{active}$. Considering the terminology explained in section 3.1, demands will be expressed as a negative value and offers as a positive one, and the objective is to leave each node in a neutral state (in this case, no demand and no offer implies a zero value) and pass the remaining demand or offer to the next listed neighbor. This is repeated for each node until the last node in the list (which is the node with the shorter $ID_{active}$, i.e., the root node) is reached. Therefore, the root will obtain the remaining offer or demand of the whole network, that is, the global balance. Afterwards, the root node, as a gateway node to the network core, might decide on its own what to do with that surplus offer or demand.

**Figure 11:**
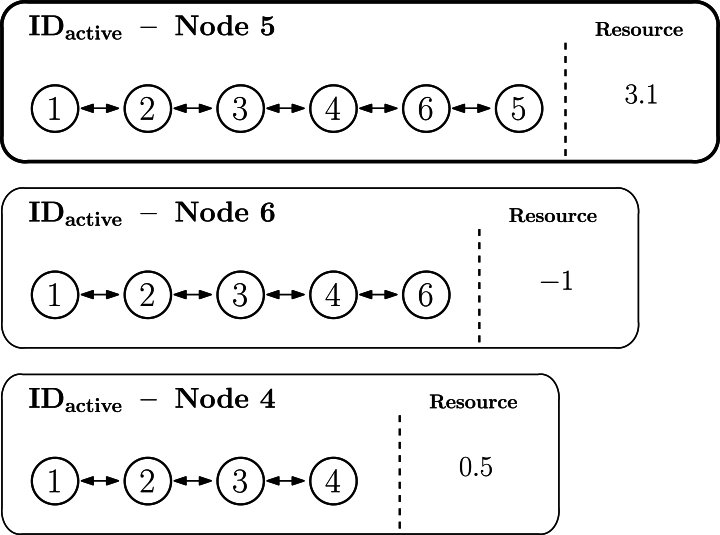
Example of the global balance procedure - step 1.

**Figure 12:**
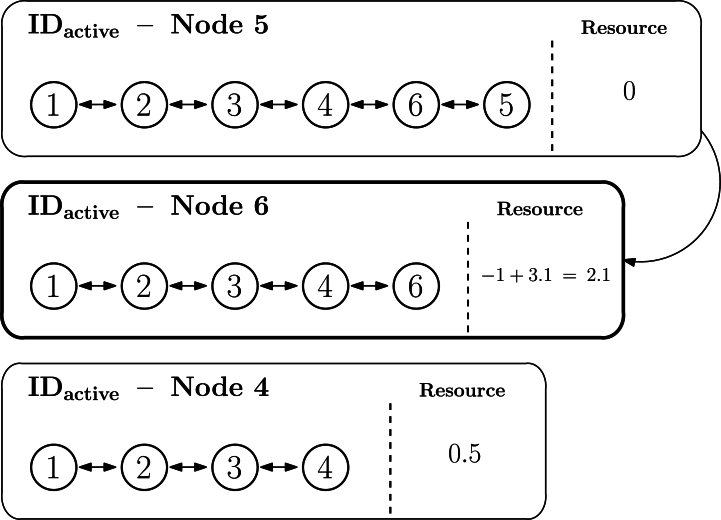
Example of the global balance procedure - step 2.

**Figure 13:**
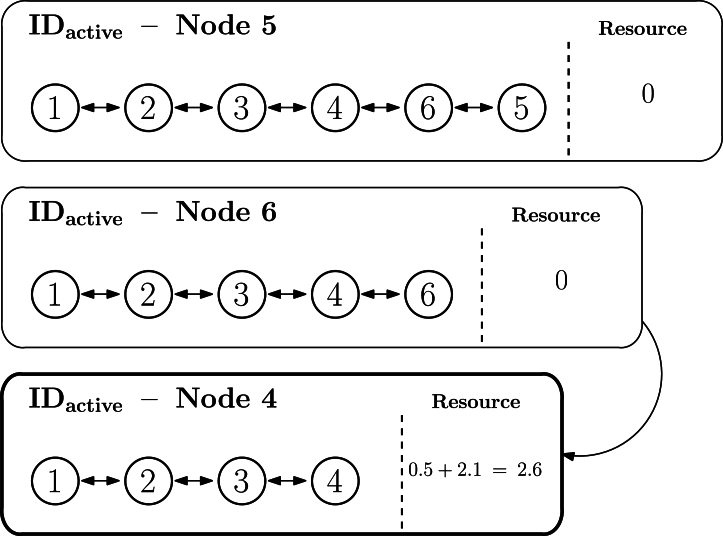
Example of the global balance procedure - step 3.

Let us exemplify the previous procedure. Fig. 11 represents the topology of the sample network used to explain the algorithm so far, in which the longest $ID_{active}$ is the one associated to node 5 (1.2.3.4.6.5). Accordingly, Fig. 11 illustrates the ordered list and the first node to be checked is node 5, while the last one is node 1. For the sake of simplicity, only the $ID_{active}$ of three first nodes to be visited (5, 6, and 4) are represented in the figure. In this scenario, node 5 has an offer of 3.1, node 6 has a demand of −1, and node 4 has an offer of 0.5. As the algorithm starts at node 5, its state will be set at zero, and its offer will pass to the next neighbor in the list, node 6, which will now update its own resources to −1+3.1=2.1, as depicted in Fig. 12. Afterwards, the next node to process will be node 6 (1.2.3.4.6), set to zero as well and its offer or demand will pass to the following node, so that node 4 is now set to 0.5+2.1=2.6, as illustrated in Fig. 13. The DEN2NE algorithm continues processing all nodes until eventually reaching the root node, so that all nodes are balanced and the root node obtains a global offer/demand value of the network.

The previous procedure is summarized in Algorithm 2. As it can be observed, the algorithm inputs are: (1) the graph G(N,L), which is the set of topology nodes (N) and links (L), and (2) the scenario_type, which can be one of the following four ones (defined only as a reference to represent, and later on evaluate, different use cases):

• **Ideal type**: It represents a scenario in which no resource transmission loss exists when the resource ($r_{i}$) is sent from a node *i* to another one *j* (i,j∈Nandi≠j) through a link, that is, $L_{ij}\;=0$. Accordingly, for any resource transmission i→j, in which the initial state is $\left\{i=r_{i},\;j=r_{j}\right\}$, the resulting resources after the transmission are $\left\{i^{\prime }=0,\;j^{\prime }=r_{i}+r_{j}\right\}$.
• **Lossy type**: It implies a loss during resource transmission, that is, L≠∅. Accordingly, for any resource transmission i→j, in which the initial state is $\left\{i=r_{i},\;j=r_{j}\right\}$, the resulting resources after the transmission are $\left\{i^{\prime }=0,\;j^{\prime }=r_{i}+r_{j}-Lij\right\}$. The loss model that will be used is described in [104], employing the transmission losses described in Equation (6).
• **Constrained link capacity type**: So far, links have no particular limitation when transmitting a resource, but if the link is constrained to certain transmission values, the resource will not be timely forwarded and the remaining will be discarded (at least, in a logical manner for the algorithm, even if the resource excess is kept at its source). More specifically, if the link capacity is $C_{ij}$, with C≠∅, and $r_{i}\geq C_{ij}$, then the associated resource transmission i→j with initial state $\left\{i=r_{i},\;j=r_{j}\right\}$ will result in the final state $\left\{i^{\prime }=0,\;j^{\prime }=\;Cij+r_{j}\right\}$. The model for the capacities of the employed links is described in [104], where it is specified that the maximum capacity of each link is $C_{max}\;=I_{max}\;\times \;V_{d}$.
• **Lossy-constrained link capacity type**: This final type merges the two previous ones, that is, L≠∅ and C≠∅. Accordingly, for any resource transmission i→j, in which the initial state is $\left\{i=r_{i},\;j=r_{j}\right\}$, the resulting resources after the transmission are $\left\{i^{\prime }=0,\;j^{\prime }=r_{i}+r_{j}-Lij\right\}$ in case $r_{i}\leq C_{ij}$, and $\left\{i^{\prime }=0,\;j^{\prime }=Cij+r_{j}-Lij\right\}$ in case $r_{i}\geq C_{ij}$.

**Algorithm 2:**
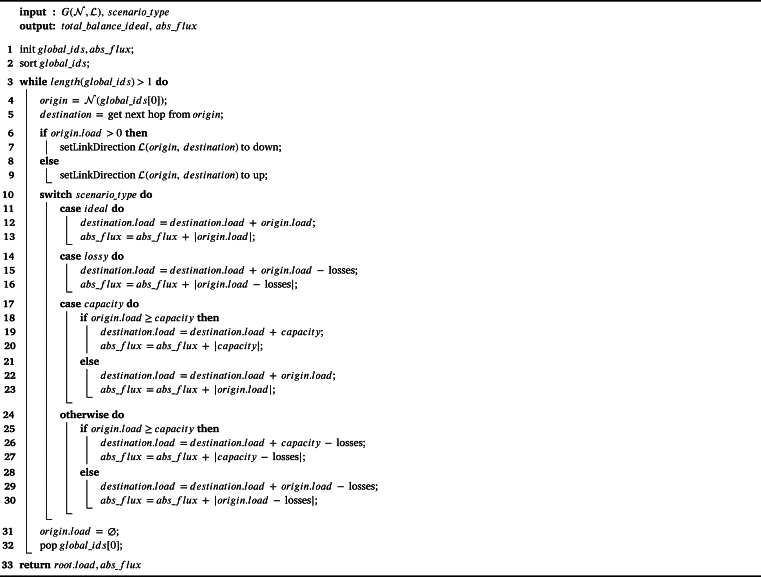
Global balance process.

Regarding the outputs of Algorithm 2, total_balance_ideal provides the overall global balance of the topology (which is indeed the root load, as it remains as the last node and keeps the difference of all resource transfer, so that is why total_balance_ideal=root.load), while abs_flux provides an absolute value of resource exchanges along the network (for any exchange, its absolute value is considered, as the negative or positive value only indicates the direction, but the resource movement occurs in any case). Therefore, total_balance_ideal expresses whether the network has an overall demand or excess of resources, while abs_flux represents the resource transfer cost. More specifically, total_balance_ideal could be equal or close to zero, which means that the network does not have a particular resource demand, but abs_flux could indicate a high value, which showcases that, in order to reach an overall balance, there is a high amount of exchanges. In fact, total_balance_ideal simply illustrates the scenario nature (i.e., amount of offers vs. demands), while abs_flux is indeed an indicator of how good the algorithm is since the lower the value, the lower is the cost to optimally balance the resource load in the topology.

As it can be observed, Algorithm 2 is linear (complexity O(n)), because network nodes are visited just once considering the selected label or ID obtained in the second phase of the algorithm, as represented by the only loop (while) in the algorithm.

Finally, it is worth noticing that the DEN2NE algorithm is executed based on a specific snapshot of the network. If the network is updated based on node mobility, or modification of offers and demands, the algorithm can be executed again, at any time, to redistribute the new resources.

## 4 Evaluation setup

The implementation and evaluation setup are based on Python 3.8.^2^ More specifically, the evaluation is divided into two stages:

1. A random topology generator to comprehensively test DEN2NE that leverages the BRITE [77] environment, as depicted in Fig. 14 (which translates the *.brite files into *.csv files for the nodes and links of the topology, used as input in our algorithm).

**Figure 14:**
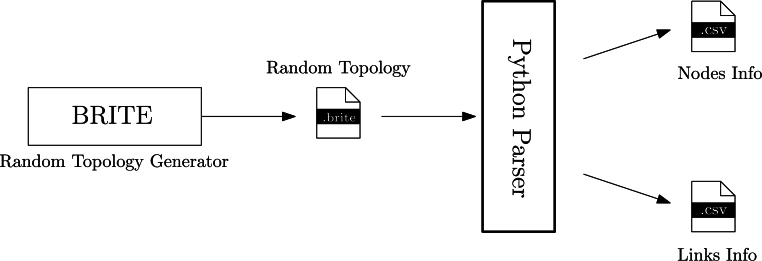
Topology generator for DEN2NE based on BRITE.
2. The execution of the algorithm, developed as a centralized script, which imports all generated topologies, one by one, using seeds and different parameters of DEN2NE for comparison.

**Figure 15:**
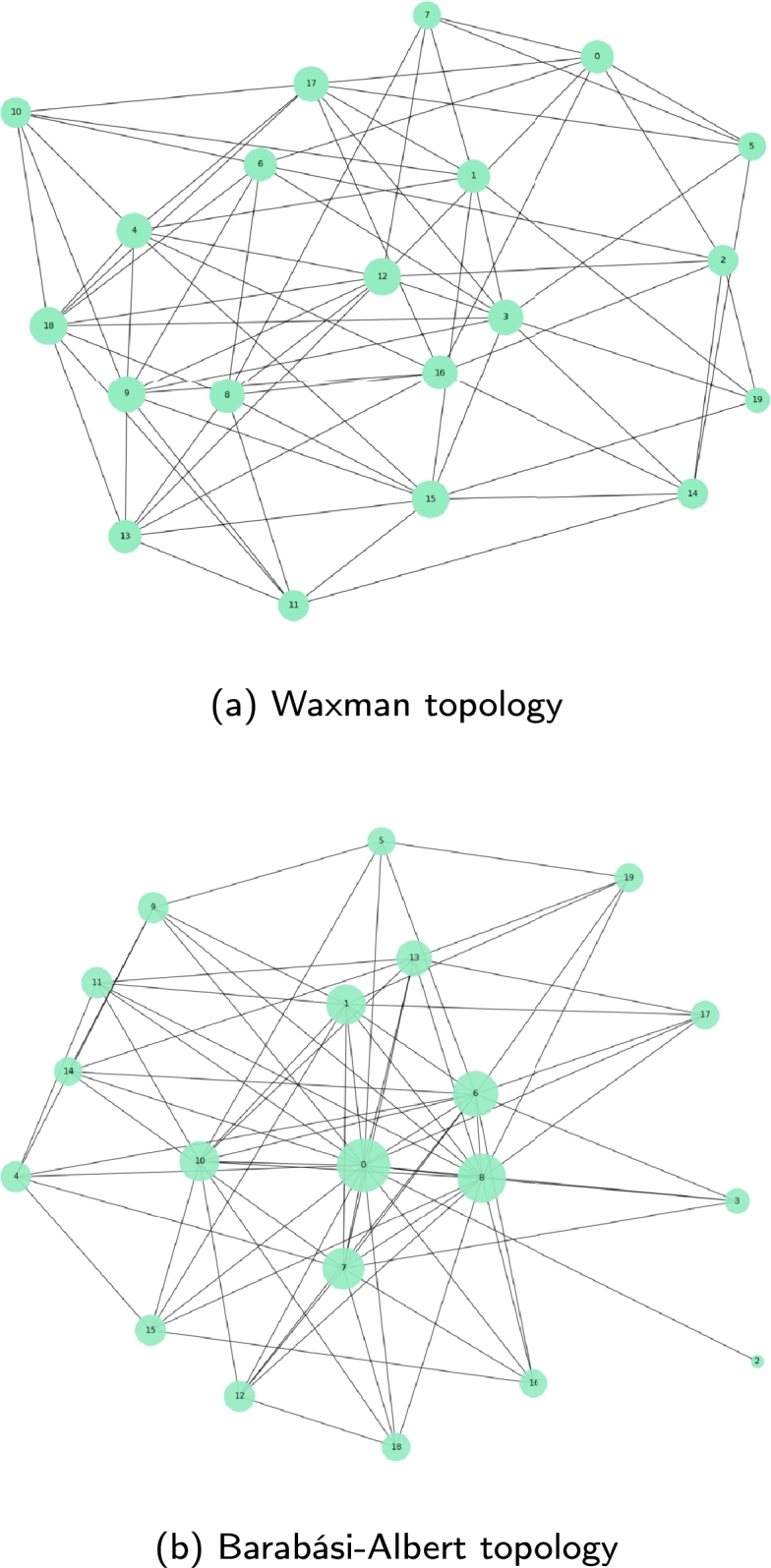
Random topologies employed in our study of DEN2NE.

Our objective is to provide a comprehensive analysis of the DEN2NE algorithm, encompassing as many types of scenarios as possible. In fact, the graphs provided by BRITE are based on Waxman [125] and Barabási-Albert [15] random topologies, which are used to model a wide range of interconnection systems, from computing networks to social networks. The Waxman random network is founded upon a probabilistic model for the random generation of a network, whereas the Barabási-Albert network falls into the category of a scale-free network (a.k.a hub-and-spoke network), in which certain nodes exhibit a significantly higher number of connections than others. A simplified view of both models is provided in Fig. 15.

Regarding the topology generation parameters, they are summarized in Table 2, which includes: topology type (one of the two types mentioned before), number of nodes (from 10 to 200 nodes in 10-node steps), connectivity degree (average number of links per node, from 2 to 6, in 2-link steps), and a random topology seed (to have 10 random topologies of each type). According to these parameters, the number of tested topologies was 1200, as indicated by Equation (14).

**Table 2:** Parameters for the generation of random topologies.

Attribute	Values
Topology type	[“waxman”, “barabasi-albert”]
Number of nodes	[10:10:200]
Connectivity degree	[2:4:6]
Random topology seed	[1:1:10]

(14) $$\langle N_{topolgies}\rangle \;=\;N_{type}\;\times \;N_{nodes}\;\times \;N_{degree}\;\times \;N_{seed}=2\times 20\times 3\times 10\;=\;1200\;\;topologies$$

**Figure 16:**
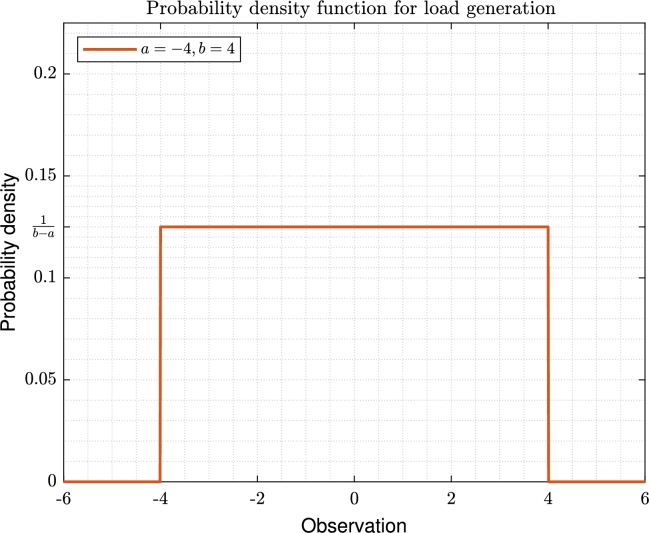
Probability density function for random load generation in each topology.

The performed evaluation aims to compare the behavior of the 6 criteria described in section 3.3 in four types of network scenarios (ideal, lossy, constrained link capacity, and lossy-constrained link capacity). Additionally, the testbed should assign an initial resource offer or demand to each topology node. This resource assignment was agnostic (no particular unit or metric is associated to it) and it could be either limited to a certain total value (which was set to 250, assuming at least 1 unit of resource per node in average in the bigger topologies, with 200 nodes) or not (no maximum value was set). Limiting the resource placement was defined to debug the algorithm implementation and behavior in the more complex scenarios. In any case, either if limited or not, the load to be placed in each node followed a uniform distribution, as depicted in Fig. 16.

Finally, to validate our results, each scenario was repeated 10 times using different seeds for the random topology generation. These seeds only changed the root location and the resource assignment, while the rest of the scenario was kept the same, to extensively prove the obtained results. All of these parameters are summarized in Table 3. Therefore, the total number of simulations of our study is 576000, as indicated by Equation (15).

**Table 3:** Parameters for test execution.

Attribute	Values
Criterions	[1:1:6]
Scenario type	[1:1:4]
Resource limitation	[0,1]
Random execution seed	[1:1:10]

(15) $$\langle N_{simulations}\rangle \;=\;N_{topologies}\;\times \;N_{criterions}\;\times \;N_{scenarios}\;\times \;N_{RL}\;\times \;N_{seed}\;\;=1200\times 6\times 4\times 2\times 10\;\;=\;576000\;\;simulations$$

### 4.1 Code repository

The implementation code of DEN2NE, as well as all auxiliary code (class hierarchy for graphs/nodes/links, etc.), is openly available at GitHub [83].

## 5 Results and evaluation

Considering the implementation and evaluation setup presented in Section 4, in this section we outline the results obtained from the described simulations.

To evaluate the behavior of DEN2NE, we measured three different metrics: the first one in relation to resource balance (which is one of the outputs from the DEN2NE algorithm, as previously described in Algorithm 2), and the other two in relation to execution time for both label assignment and resource balance, as our objective was to assess how good is DEN2NE to balance the load and how fast it is to perform that task.

For this reason, the following three sections examine these parameters in detail, considering the network size, type and connectivity, the scenario type, and the six criteria defined in section 3.3. All metrics have been performed with multiple repetitions, as stated in Section 4, and all include their respective standard deviation though it is negligible in most graphs.

It is important to note that the validity of the results obtained in this section is limited to the implementation conducted in Section 4. In practice, if the scenario includes a central software controller, we might obtain very similar results. However, for instance, the communication times from the controller to the other nodes involved in the network would need to be added, which would depend on conditions entirely unrelated to the algorithm itself.

### 5.1 Resource balance flow

The resource balance flow represents the amount of resources moved among all topology nodes. This metric has been calculated by summing up all resource exchanges between pairs of nodes as an absolute value, and it was previously presented in Algorithm 2 as the variable *abs_flux*. This metric indicates the expense of balancing resources: the higher the value, the costlier is this balance.

**Figure 17:**
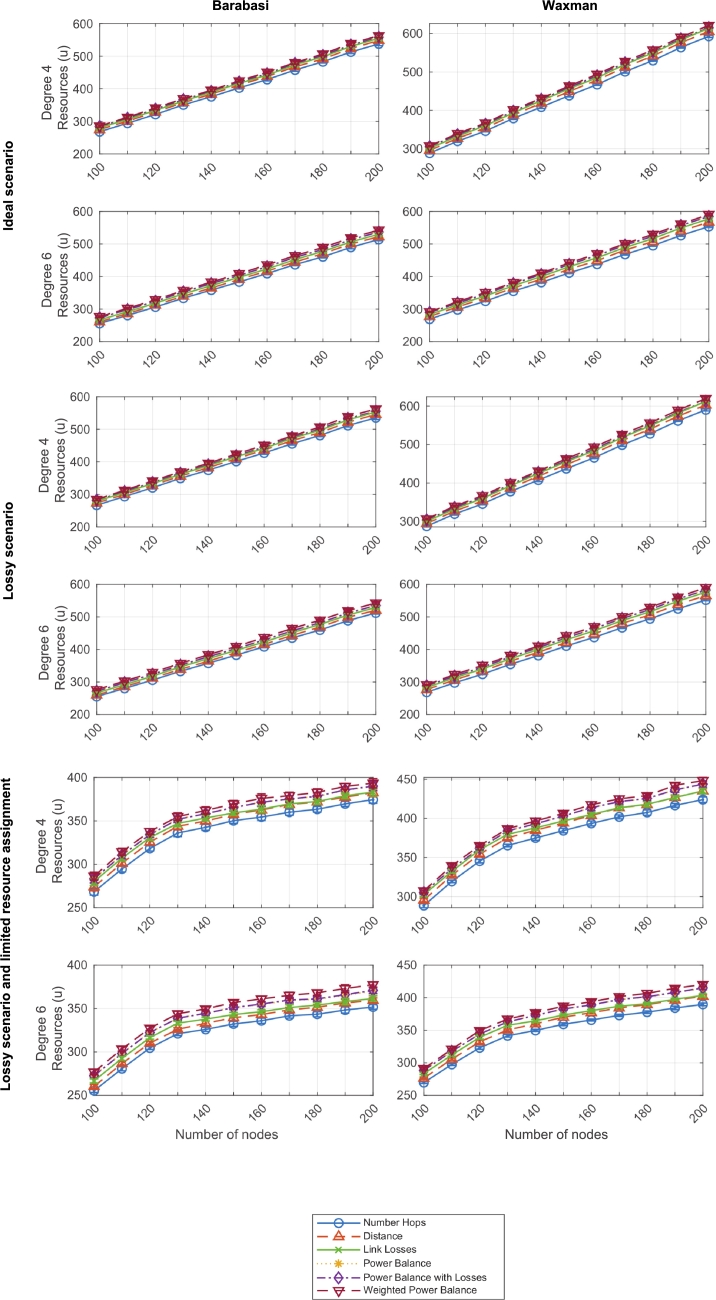
Resource balance flow (*abs_flux*) - Three scenarios and two network types (degrees 4 and 6).

Fig. 17 illustrates the resource balance flow for networks with connectivity degrees from 4 to 6 based on Barabasi and Waxman topologies from 100 to 200 nodes, and tested with the six defined criteria, in three types of scenarios: ideal, lossy, and lossy with resource assignment limitation, respectively.

As it can be observed, the flow linearly grows with topology size, which indicates that the algorithm presents a scalable behavior (O(n)), not severely influenced by the amount of nodes involved. Only the last scenario, represented by the four graphs located at the bottom of Fig. 17, portrays a logarithmic growth, but this effect is due to the resource assignment limitation.

Additionally, considering the six criteria, it seems that Criterion 1 (number of hops) yields better results (smaller flow) than the rest, particularly in Waxman topologies and when node connectivity increases; while Criterion 6 (weighted power balance) seems to be the worst option, regardless of the scenario (even if lossy or resource-constrained). The discrepancy between these criteria can be explained by the fact that criteria aimed at minimizing distance or hops to the root node favor shorter paths, while criteria that consider the quantity of resources along the path to the root tend to select longer paths. Therefore, in Criterion 1 and Criterion 2 (number of hops and distance, respectively), the average resource flow is lower compared to Criterion 3 and Criteron 6 (power balance and weighted power balance, respectively), where paths will be longer on average, resulting in a higher resource flow.

### 5.2 Label assignment convergence time

Label assignment convergence time represents the time required by the algorithm to assign all hierarchical labels from the root to the rest of topology nodes.

**Figure 18:**
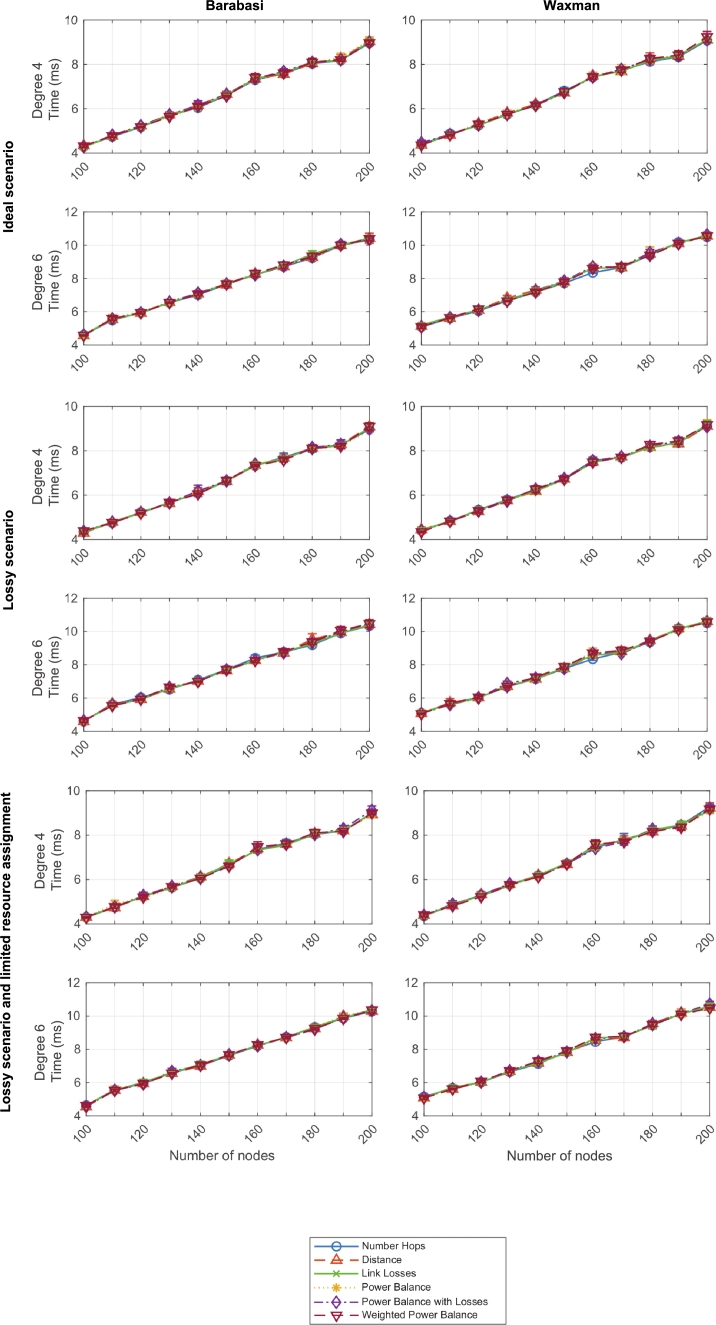
Label assignment convergence time - Three scenarios and two network types (degrees 4 and 6).

Fig. 18 illustrates the label assignment convergence time for networks with connectivity degrees from 4 to 6 based on Barabasi and Waxman topologies from 100 to 200 nodes, and tested with the six defined criteria, in three types of scenarios: ideal, lossy, and lossy with resource assignment limitation, respectively.

As it can be observed, this convergence time linearly grows with the topology size, and it is not exponentially affected by connectivity degrees or other parameters. This fact outlines a potentially good scalability of the algorithm as, even if the worst-case scenarios, the convergence time does not surpass 15 ms. Only Waxman topologies of degree 2 yield a relatively high standard deviation (in comparison to other results, and probably due to outlier values as the connectivity is low), but still below the 1 ms convergence time.

Furthermore the six criteria present really close behaviors, without clear differences. This is because this label assignment convergence time strictly depends on the first phase of the algorithm, which is independent from the applied criterion afterwards. If any differences, those might be due to the random nature of the performed tests.

### 5.3 Resource balance convergence time

Resource balance convergence time represents the time required by the algorithm to perform all the resource exchanges so that the topology is balanced up to the root.

**Figure 19:**
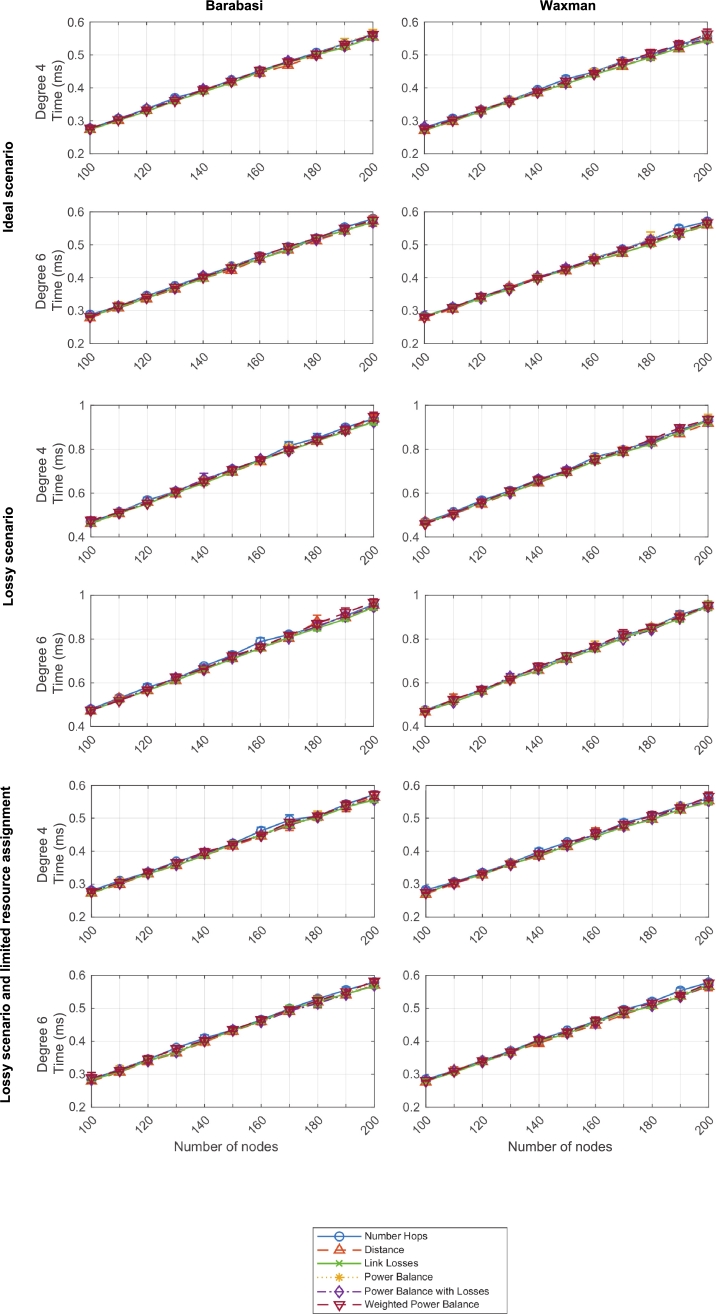
Resource balance convergence time - Three scenarios and two network types (degrees 4 and 6).

Fig. 19 illustrates the resource balance convergence time for networks with connectivity degrees from 4 to 6 based on Barabasi and Waxman topologies from 100 to 200 nodes, and tested with the six defined criteria, in three types of scenarios: ideal, lossy, and lossy with resource assignment limitation, respectively.

As it can be observed, and similarly to the previous label assignment convergence time, the resource balance convergence time linearly grows with the topology size, and it is not exponentially affected by connectivity degrees or other parameters. This fact outlines a potentially good scalability of the algorithm as, even if the worst-case scenarios, the convergence time does not surpass 1 ms. Therefore, the total execution time of the DEN2NE algorithm was shorter than 20 ms in all evaluated scenarios.

Moreover, as in the previous evaluated time measurement, the six criteria showcase a very similar behavior. In this case, resource balance convergence time does depend on the six criteria (not as the previous one), but still the algorithm definition has a bigger impact on this time that the selected criteria. This is particularly relevant because criteria are defined to match better resource allocation in different scenarios. Therefore, these results demonstrate that our algorithm can be adjusted and tailored to specific use cases without suffering meaningful changes in execution time.

## 6 Discussion

After a comprehensive evaluation in terms of balance flow and convergence time, with multiple network topologies and objective criteria, results showcase that DEN2NE exhibits a linear growth with the network size, independently of the network type. Furthermore, convergence times do not exceed 15 ms in the most complex cases, even considering our algorithm was not specifically optimized in terms of execution time when coded (it is important to note that these results were obtained based on a centralized execution of the algorithm developed as a Python script, under the conditions described in Section 4). Moreover, DEN2NE is a generalized algorithm that can be adjusted to certain scenarios based on one of the six criteria or any additional one, if required. This grants the possibility to configure a very short period for updates (if needed). Accordingly, DEN2NE is particularly suitable for environments with frequent updates or high mobility, as DEN2NE can rapidly incorporate new information and refresh actions and decisions based on it. For instance, scenarios in which resilience is critical might benefit from the speed of our algorithm.

On the other hand, DEN2NE follows a centralized approach, which might not be feasible to implement in all types of networks. Moreover, the current evaluation is based on wired networks only; while wireless networks could also be evaluated, additional parameters such as mobility or resilience should be analyzed. Finally, for the sake of simplicity, we have evaluated DEN2NE in single-root scenarios, although it could also work in multiple-root ones, and that could be examined in detail as well.

In the following paragraphs, we discuss potential fields of application for DEN2NE, considering the current state of the art.

### 6.1 Use cases and fields of application

After a comprehensive analysis of uses cases, we have found three main potential fields of application for DEN2NE: microgrids, edge computing, and last-mile delivery. In the next sections, we examine related works and how DEN2NE could contribute to the state of the art.

#### 6.1.1 Microgrids

In the context of electricty systems, a microgrid is a small-scale, localized energy system that can operate independently or in conjunction with the main power grid [115]. It typically includes diverse energy resources, such as renewable energy sources (e.g., solar panels) or energy storage systems (e.g., batteries). The microgrid nodes can be either consumers or producers, or both, i.e., *prosumers*. Microgrids facilitate the distribution of energy, providing resilience and flexibility to traditional power grids. Furthermore, they can either operate autonomously or in coordination with the main grid. Therefore, microgrids are often good solutions for remote locations or residential areas, which could work as *energy islands* if needed.

Although microgrid networks are mainly static, they rely on switches to activate and deactivate parts of the network (usually meshed in this context, to grant redundancy) in order to adjust electrical distribution. In that sense, microgrids currently lack effective mechanisms to update that distribution (that is, to decide what switches should be turned on and off to maximize flow distribution) in a fast and optimal manner [113], and usually follow manual or traditional procedures. For example, load redistribution is required for service restoration [20], but few works explore energy routing [98], [67], [101], [53], [97], [33], [59], [116], which might be particularly needed in networked microgrids [44], [103], when several microgrids are interconnected to augment resilience.

In this field of application, DEN2NE could be leveraged for dynamic updates for energy load distribution (i.e. to activate and deactivate switches, for instance), particularly for events that require fast reactions, such as cyberattacks [120]. Current proposals do not scale well with big networks and they require minutes for redistribution and/or a previous training with data to apply optimization techniques, but DEN2NE is able to grant a faster recovery.

#### 6.1.2 Edge and fog computing

In the case of communication networks, moving the intelligence of the network to the edge has clear benefits, but distributing that capacity among the diverse network nodes is not straightforward [12].

Most related works rely on AI/ML techniques for this task [65], [8], [82], [74], although game theory is also leveraged [17], [32]. However, the most relevant aspect is that few works explore the option of a multi-hop resource sharing mechanism and for very specific scenarios, such as UAV and vehicular networks [16], [48], [30], [21], [61], [52], [117], [46], [29], [4], [47]. Furthermore, most of them do not implement mechanisms for reconfiguration after network changes/updates and/or requiere long times of calculation, what constrains its potential application to dynamic scenarios with high mobility.

In this scenario, DEN2NE facilitates fast reconfiguration times, as its convergence time does not exceed 20 ms in total in networks of up to 200 nodes (with just a single gateway) and exhibits a linear growth with bigger networks. Furthermore, its light implementation particularly suits the constrained user equipment involved in edge and fog computing.

#### 6.1.3 Last-mile delivery

During the last decade, last-mile delivery has boomed as a cornerstone of logistics [106]. Furthermore, crowdshipping (demand and supply of goods by crowd with available capacity) [25] is targeted as a pillar for sustainable urban logistics [111]. This use case resembles the two previous ones in the sense that demands of resources (which in this context would be transport means) might be fulfilled by any available source in the network. As an example, when a collection of packages from various companies is destined for the same recipient or recipients located in close proximity, it is inefficient for each package to be delivered by a separate logistics provider. This practice frequently results in unnecessary time expenditure and increased environmental pollution. Therefore, it is advisable to coordinate the delivery process to allow for the consolidation of packages among different logistics companies as needed. In this regard, logistical resources should be optimized, and this is where algorithms like DEN2NE come into play.

Diverse proposals exist in this area, involving goods like, for instance, parcels [35], food [66], [84], passengers [112], or a combination of these [19]. In any case, the resource to be shared is transportation (e.g. represented as a fleet [71], [70], [51], [80] or even as public transport [24] Once again, current proposals in the state of the art rely on optimization techniques and AI/ML [118], [107], [31], [127], [112], [80], but some related works also leverage auction-based approaches [64].

As last-mile delivery is a high-mobility use case, DEN2NE could clearly accelerate decision making by dynamicly updating scenarios every few seconds and providing the best resource sharing at each time. We also believe that additional criteria could be designed based on the specific requirements of this field of application, which might involve different factors (such as fast deliveries, user satisfaction, user tailored delivery times, fleet comfort and pause times, or transport pollution).

#### 6.1.4 Other use cases

Flow control in natural gas networks represents an alternative use case in which this algorithm could be applied [137], similarly to electricty networks. However, these networks are usually more static and might not be so benefited from the application of DEN2NE.

WiFi channel assignment is also another potential field of application [75], in which nodes need to individually decide their WiFi channel based on collective intelligence to improve the exploitation of available resources.

## 7 Conclusion

In this manuscript, we have presented DEN2NE, an algorithm for resource management in multi-hop dense networks that exhibits great scalability and low convergence times.

The algorithm discovers the different nodes in the topology and assigns one or more hierarchical labels to each node, which represents multiple paths to the root or gateway node. Afterwards, it reassigns workload from the nodes located in the lower part of the hierarchy until the higher ones. This reorganization of resources can follow different criteria, and we presented six different types as potential examples, but it could be extended with any other criterion, as the algorithm definition is not constrained and can be tailored based on the actual application scenario.

The evaluation was performed in a wide range of topologies, up to 200 nodes, and results exhibit a short convergence time (lower than 20 ms) that follows a linear growth. This clearly benefits scenarios with high mobility and/or that require augmented reliability. Moreover, the label assignment procedure only requires two steps for execution and nodes only need to save a number of labels equal to the number of paths towards the root, independently of network size. Therefore, its implementation in nodes with constrained memory or battery is possible.

Finally, we briefly discuss its main features and potential next steps of development, together with the description of use cases, focusing on three that could leverage DEN2NE for their resource management decisions: microgrids, edge and fog computing, and last-mile delivery.

## CRediT authorship contribution statement

**David Carrascal:** Writing – review & editing, Writing – original draft, Visualization, Validation, Software, Investigation, Formal analysis, Conceptualization. **Elisa Rojas:** Writing – review & editing, Writing – original draft, Validation, Supervision, Resources, Project administration, Methodology, Investigation, Funding acquisition, Formal analysis, Conceptualization. **Juan A. Carral:** Writing – review & editing, Supervision, Project administration, Methodology, Investigation, Funding acquisition, Formal analysis, Conceptualization. **Isaias Martinez-Yelmo:** Writing – review & editing, Supervision, Resources, Project administration, Investigation, Funding acquisition. **Joaquin Alvarez-Horcajo:** Writing – review & editing, Validation, Supervision, Software, Investigation, Formal analysis, Conceptualization.

## Declaration of Competing Interest

The authors declare that they have no known competing financial interests or personal relationships that could have appeared to influence the work reported in this paper.

## Data Availability

The algorithm implementation and associated data is publicly available in GitHub in the following URL: https://github.com/NETSERV-UAH/den2ne-Alg.

## Footnotes

1 It is important to highlight the adverb *logically* in this context, as the node might be physically located closer, but the selected $ID_{active}$ might provide a different logical view.

2 https://peps.python.org/pep-0569/.
